# An assessment of trends in the frequency and duration of *Karenia brevis* red tide blooms on the South Texas coast (western Gulf of Mexico)

**DOI:** 10.1371/journal.pone.0239309

**Published:** 2020-09-18

**Authors:** Sarah A. Tominack, Kara Z. Coffey, David Yoskowitz, Gail Sutton, Michael S. Wetz

**Affiliations:** Harte Research Institute for Gulf of Mexico Studies, Texas A&M University – Corpus Christi, Corpus Christi, Texas, United States of America; Mississippi State University, UNITED STATES

## Abstract

Limited data coverage on harmful algal blooms (HABs) in some regions makes assessment of long-term trends difficult, and also impedes understanding of bloom ecology. Here, observations reported in a local newspaper were combined with cell count and environmental data from resource management agencies to assess trends in *Karenia brevis* “red tide” frequency and duration in the Nueces Estuary (Texas) and adjacent coastal waters, and to determine relationships with environmental factors. Based on these analyses, the Coastal Bend region of the Texas coast has experienced a significant increase in the frequency of red tide blooms since the mid-1990s. Salinity was positively correlated with red tide occurrence in the Nueces Estuary, and a documented long-term increase in salinity of the Nueces Estuary may be a major factor in the long-term increase in bloom frequency. This suggests that freshwater inflow management efforts in Texas should consider impacts on red tide habitat suitability (i.e., salinity regime) in downstream estuaries. Natural climate variability such as the El Niño-Southern Oscillation, which is strongly related to rainfall and salinity in Central and South Texas, was also an influential predictor of red tide presence/absence. Though no significant change in the duration of blooms was detected, there was a negative correlation between duration and temperature. Specifically, summer-like temperatures were not favorable to *K*. *brevis* bloom development. The relationships found here between red tide frequency/duration and environmental drivers present a new avenue of research that will aid in refining monitoring and forecasting efforts for red tides on the Texas coast and elsewhere. Findings also highlight the importance of factors (i.e., salinity, temperature) that are likely to be altered in the future due to both population growth in coastal watersheds and anthropogenic climate change.

## 1. Introduction

Red tides formed by the marine dinoflagellate *Karenia brevis* have affected Gulf of Mexico coastlines for centuries [1, 2], typically during late summer-fall. The most notable effects are fish kills, shellfisheries closures, marine mammal and seabird mortality, and respiratory and digestive distress in humans [3–5]. In the United States, the West Florida and Texas coastlines are most commonly affected, with Florida historically suffering the most damage [1, 5–7]. An analysis of trends in *K*. *brevis* red tide occurrence was conducted for Florida coastal waters and indicated that frequency of occurrence, intensity, and duration were higher in the years 1994–2002 compared to 1954–1963 [2]. Magaña et al. [1] reported that the frequency of red tides on the Texas coast increased over the period of 1996–2000 compared to earlier years. In both instances, availability of historical data limited the scope of inferences that could be drawn from study findings [8–10].

Eutrophication is often cited as the cause of increases in harmful algal blooms (HABs) globally [11–13]. In the case of *K*. *brevis* however, its ability to use nutrients from a wide variety of sources has called into question the role of eutrophication as the main factor causing increased bloom frequency and intensity [10, 14]. In Florida for example, studies suggest that a complex suite of environmental conditions determine bloom formation. Briefly, downwelling conditions followed by upwelling concentrates *K*. *brevis* and subsequently transports it shoreward [6, 15, 16]. During transport, *K*. *brevis* is thought to acquire nutrients from sediment porewater (directly or via benthic flux), zooplankton excretions, bacterial remineralization, upwelled deep-water nutrients, and “leaky” *Trichodesmium* blooms in an otherwise oligotrophic environment [9, 10, 14]. It is only in the nearshore and estuarine environments where blooms come into contact with relatively high nutrient waters [9, 10, 14].

In Texas, physical concentration and advection of cells is also important in the initiation of red tides. Recent modeling work suggests a southern origin of red tides and a general transport pattern of: 1) summer upcoast winds carry seed populations from the southern Gulf of Mexico to the Texas coast, and 2) a switch to downcoast winds from summer to fall that produce Ekman transport towards the coast, delivering *K*. *brevis* to the near-shore environment [17–19]. Though physical mechanisms are critical in the development/transport of *K*. *brevis* in West Florida and Texas blooms, environmental conditions in coastal waters must also be suitable. Field and laboratory studies have consistently demonstrated strong relationships between *K*. *brevis* and salinity and temperature, with higher salinities (20–40) and low to moderate temperatures (7°C—32°C) related to greater *K*. *brevis* success [14, 20, 21]. Blooms in Texas are frequently transported into estuaries [22], and there is also anecdotal evidence of blooms developing within the estuary as opposed to coastal waters [23]. Unfortunately, there have been no studies to date on *K*. *brevis* population dynamics in Texas estuaries. Additionally, despite occurrences of *K*. *brevis* red tides in Florida estuaries on the Gulf and Atlantic Coasts [24–28], few have addressed questions regarding *K*. *brevis* ecology in an estuarine setting [20, 29, 30], highlighting a critical gap in our knowledge.

A major challenge for assessing trends in the environmental sciences is the lack of long-term data [13, 31]. Nonetheless, since the early 2000’s significant advances have been made by utilizing non-traditional data sources, resulting in emergence of a new field, marine historical ecology [32, 33]. Successful case studies have used newspaper articles, diaries, correspondence, photographs, and maps to reconstruct historical fisheries populations and ranges, assess loss of historical ecosystem services, and set ecosystem restoration targets [32, 33]. Here we combined information on red tide occurrences from local news articles with cell count data from resource management agencies to assess long-term trends in red tide frequency and duration in the Nueces Estuary (Texas) and adjacent coastal waters. The goals of this study were to: 1) extend the temporal record of red tides in a portion of the Texas Coastal Bend using validated newspaper accounts, 2) quantitatively assess trends in red tide frequency in a data poor region (estuarine/nearshore waters of the South Texas coast) and environmental factors associated with red tide occurrence, and 3) use these data to increase understanding of *K*. *brevis* red tide dynamics in an estuarine setting. This is important because estuaries of the South-Central Texas coast are undergoing significant environmental change due to rapid population growth and climate change [34]. Furthermore, climate scenarios suggest that the region will become hotter and drier in the future [35, 36]. Results from this study offer insight into the utility of non-traditional data for detection of long-term trends and red tide population dynamics in a data poor region (Texas coast). Additionally, these results can be used to inform monitoring programs, improve predictive capabilities, and to develop targeted studies to address key questions regarding *K*. *brevis* ecology.

## 2. Materials and methods

**Fig 1:**
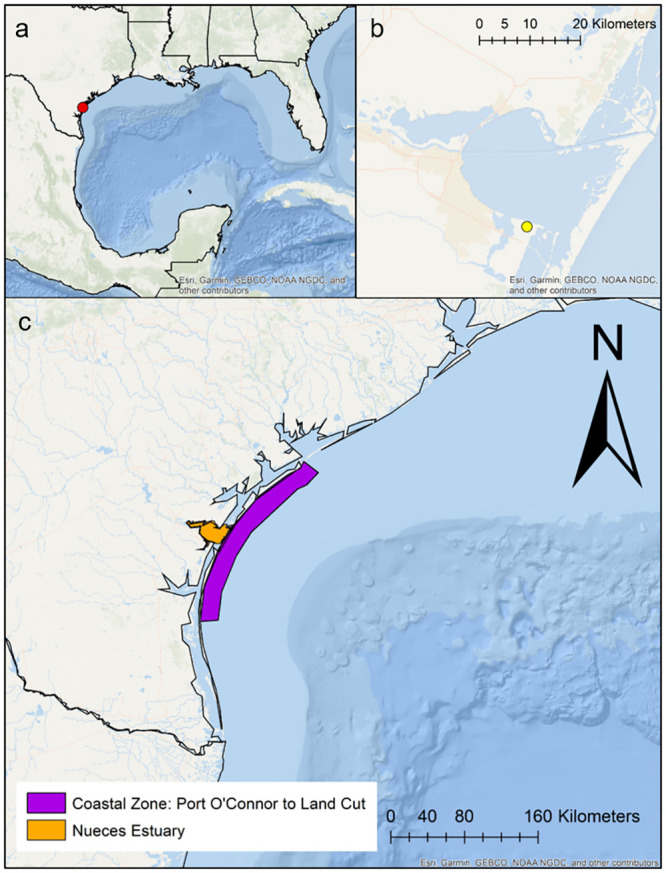
Map of study areas on Texas Gulf of Mexico coastline. (A) location of Nueces Estuary in the Gulf of Mexico (red circle), (B) zoomed in view of the Nueces Estuary and the location of Naval Air Station Corpus Christi (yellow circle), and (C) location of the Nueces Estuary relative to the adjacent coastal zone. The coastal zone segment (purple) extends from the Land Cut in the south to Port O’Connor in the north.

Daily newspaper articles from *Corpus Christi Caller Times* from 1955 through 2016 were obtained and read for relevant articles on red tide. Information from the newspaper articles was then aggregated into yearly presence/absence and duration (days) datasets for each of two segments, the Nueces Estuary and the coastal zone from Port O’Connor to Land Cut (Fig 1).

In cases when a single red tide spanned two calendar years, its presence and total duration would only be recorded in the first year. For example, if a bloom began in September of Year 1 and ended in January of Year 2 the total duration in days across both years was recorded as the duration for year 1.

Cell counts of *K*. *brevis* were obtained from the NOAA repository (2005–2013; https://data.nodc.noaa.gov) [37] and the Texas Department of Health Division of Seafood Safety (1996–2016; TXHD). Each cell count record was converted to categorical presence/absence. A comparison between the newspaper and agency presence/absence and duration data was conducted using three cell count thresholds from the agency data: 5,000 cells L^-1^ (shellfisheries closures), 10,000 cells L^-1^ (fish killing levels), and 100,000 cells L^-1^ (visual detection likely) [6, 20].

### 2.1 Accessory data collection

Bay-wide salinity and temperature data (monthly) were obtained from the Texas Parks & Wildlife trawl sampling dataset (S1 Table). Monthly climate indices for the El Niño Southern Oscillation (ENSO), the Pacific Decadal Oscillation (PDO), and the North Atlantic Oscillation (NAO) were accessed through NOAA’s National Climatic Data Center (https://www.ncdc.noaa.gov). Daily meteorological data were also accessed through NOAA’s National Climatic Data Center, using a weather station located at the Naval Air Station, Corpus Christi (Fig 1). Average daily air temperature, precipitation, and wind speed were chosen as the variables of interest because of known linkages between these indicators and *K*. *brevis* [6, 9, 38]. Air temperature was used as a proxy for water temperature due to the relatively short and intermittent water temperature record (1995–1996; 2006-present) available for the study area. Air and water temperature showed a strong linear relationship, though at air temperature <15°C the relationship was not as strong (S1 Fig). However, these cooler temperatures are outside the typical seasonal window for *K*. *brevis* in Texas.

### 2.2 Statistical analysis

The following analyses were conducted in R v 3.6.2. The associated code can be found in the GRIID-C data repository at http://doi.org/10.7266/7VRN6BXA.

#### 2.2.1 Trends in bloom presence/absence & relationship with environmental factors

Logistic regression (LogisticDx v 0.2) [39] was used to explore trends in red tide occurrence, and relationships with environmental variables. Year was used as the sole explanatory variable to assess changes in the frequency of red tide occurrence for the Nueces Estuary and adjacent Coastal Zone. To better understand any changes detected with the year-only logistic regression, non-parametric change point detection was performed using Pettitt’s Test for both locations (trend v. 1.2.2) [40, 41]. Yearly averages of ENSO, NAO, PDO, and fall (Aug-Nov) averages of water temperature and salinity were used, in addition to year, to assess the influence of environmental variability on red tide occurrence in the Nueces Estuary and the Coastal Zone. No collinearity was detected among the regressors, and all variables were used in creation of the initial full model. Dredge and a summary of model averages (MuMIn v 1.43.6) [42] were used to determine the importance of each explanatory variable and the best models were built. The models were compared for relative quality using Akaike’s Information Criterion (AICc) and assessed for goodness of fit using the Hosmer-Lemeshow test (LogisticDx v 0.2) [39]. The year-only and final explanatory models were compared to the null model (only presence/absence and the intercept included) as a final check for model suitability. Nagelkerke pseudo R^2^ values (pscl v 1.5.2) [43] were also calculated to assess the variability explained by each of the models and can be interpreted similarly to a traditional R^2^ value [44, 45]. Finally, the odds ratios for each variable in the year-only and final explanatory model were calculated to aid in the interpretation of the influence of each variable. For the year-only model, the odds ratio is interpreted as the probability of a red tide occurring for each step forward in time, e.g. an odds ratio of 1.17 indicates that there is a 17% increase in the probability of a red tide occurring with each passing year. For the explanatory models, the odds ratio is not interpreted in the same way due to differing scales among the explanatory variables, optimum temperature and salinity ranges of red tide growth/survival, and the multivariate nature of the models. Instead, the odds ratios are presented in the results section as a metric to aid in the full appreciation of the model output. For multivariate models, the odds ratio is interpreted as the likelihood of a red tide occurring due to a change in one variable with all other variables held constant, e.g. an odds ratio of 1.17 for variable a indicates that, with all else held constant, there is a 17% increase in the probability of a red tide occurring with each 1 unit increase in variable a. If model fit, pseudo R^2^, AICc, and/or comparison to the null model was not acceptable, the analysis concluded with the year-only model.

#### 2.2.2 Trends in bloom duration & relationship with environmental factors

Linear regression was used to explore trends in *K*. *brevis* red tide duration in the Nueces Estuary and adjacent Coastal Zone with year as the only explanatory variable. To explore relationships between environmental parameters and red tide duration, models with water temperature, salinity, NAO, PDO, and ENSO (described in section 2.1) were used to create an initial full linear regression model. Dredge and a summary of model averages (MuMIn v 1.43.6) [42] were used to determine the importance of each explanatory variable and the best models were built. The models were compared for relative quality using AICc and assessed for goodness of fit. The final model was compared to the null model (only duration and the intercept) as a check of model suitability. If model fit, pseudo R^2^, AICc, and/or comparison to the null model was not acceptable, the analysis concluded with the year-only model.

#### 2.2.3 Environmental conditions associated with bloom stages

In a given year, each day that red tide was present was assigned a “1” and a two-week (14 days) buffer of “0s” was assigned before the red tide was detected (as reported in news articles) and after detection ceased. These three periods were coded as before bloom (B), during bloom (D), and after bloom (A) stages. As a part of initial data exploration, ANOVAs were used to compare average daily air temperature, precipitation, and wind speed among the three bloom stages. To account for interannual variation and seasonal variation, year and month were included as random factors. A total of three datasets were used in the following analyses: the original daily presence/absence containing all three stages, daily presence/absence dataset containing only B and D stages, and daily presence/absence dataset containing only D and A stages. Following this, generalized linear mixed-effects logistic regression (lme4 v 1.1–20) [46] was used to model daily red tide presence/absence on the three datasets. Dredge and a summary of model averages (MuMIn v 1.43.6) [42] were used to determine the importance of each variable. The best models were built and compared for relative quality using AICc and assessed for goodness of fit. The final model chosen for each dataset was compared to the null model (only presence/absence and the intercept included) as a final check for model suitability. Additionally, Nagelkerke pseudo R^2^ values were calculated for each of the three models to assess the variability explained.

## 3. Results

### 3.1 Validation of the *Corpus Christi Caller Times* dataset

Qualitative analysis of the newspaper and agency-derived cell count datasets provided evidence that the newspaper reports were a reliable source of information on the occurrence and duration of red tides in the Texas Coastal Bend. Beginning with the time frame covered by NOAA (2005–2013) and/or TXHD (1996–2016) cell counts, there was near perfect corroboration of annual red tide occurrence reported in the newspaper (S2 Table). For the Nueces Estuary, there was one instance (2012) of a red tide being reported in the newspaper that was not in either of the agency records. The newspaper article describing the 2012 red tide does, however, quote Texas Parks and Wildlife scientists on the location of the red tide “drifting in Corpus Christi Bay through the Port Aransas Jetties” lending support to it being a real occurrence that was not documented by agency-based sampling. There were no instances of red tides reported in the agency record that were missed by the newspaper. In the Coastal Zone there were two instances (2000, 2012) of a red tide being reported in the newspaper that did not appear in the TXHD or NOAA records. The red tide occurrence in 2000 is, however, corroborated by a study by Cheng et al. [40], while the single day event reported in the coastal zone in September 2012 is from the same article mentioned above where observations were corroborated by state resource managers. There was also one instance of a red tide reported in the scientific record but not in the newspaper (2013) for the coastal zone during this time.

Prior to 1996, there is not a unified agency database for comparison. Five of the eight red tides that were reported in the newspaper to have occurred between 1955 and 1995 along the Texas coast were, however, captured in the literature (Table 1) [1, 22, 47–49].

**Table 1:** Summary of *Corpus Christi Caller Times* articles addressing red tides. The start and end dates of blooms reported in articles is indicated for the Nueces Estuary and adjacent Coastal Zone. References for published work that corroborates occurrence of red tides prior to 1996 appear in the ‘Notes’ column along with other relevant information. NA is not applicable, NR is not reported.

Year	No. Articles	Region Affected	Start- Nueces	End- Nueces	Start- Coastal Zone	End- Coastal Zone	Notes
1955	1	Texas coast near Mexico border	NA	NA	NA	NA	Wilson and Ray [49]
1957	1	Tampico, Mexico	NA	NA	NA	NA	Discusses current diatom bloom in Coastal Bend and red tide work by US Fish and Wildlife Service
1963	2	West Florida	NA	NA	NA	NA	Discusses current scientific knowledge of red tides and previous occurrences in Texas in 1934, 1955, and 1874, the latter not authenticated.
1970	3	Nueces Estuary	7/6/1970	7/10/1970	NA	NA	
1972	2	Coastal Bend	NA	NA	10/25/1972	NR	Description of red tide in New England; Texas Parks and Wildlife data cited in Magaña et al [1]
1973	4	Coastal Bend	5/4/1973	5/4/1973	5/4/1973	5/4/1973	
1974	4	Mexico	NA	NA	NA	NA	Describes wind as potential factor for red tide to move north and work in Florida to predict red tides; Texas Parks and Wildlife data cited in Magaña et al [1]
1975	5	Nueces Estuary	8/6/1975	8/9/1975	NA	NA	Latter three articles describe other red tide on Upper Texas Coast not attributed to *K*. *brevis*
1980	2	Nueces and Lavaca-Colorado Estuaries	NA	NA	NA	NA	Describes discolored water and fish kills attributed to a chemical spill and an organism other than K. brevis, respectively
1986	71	Texas coast	10/8/1986	1/12/1987	9/7/1986	10/25/1986	Trebatoski [48]
1987	13	NA	NA	NA	NA	NA	Describes the effects of the red tide from the previous year and potential disaster relief funding
1988	2	Puget Sound, Washington, USA	NA	NA	NA	NA	Describes red tides in general, the 1986 red tide in Texas, and a red tide occurring in Puget Sound
1990	4	Mission Aransas Estuary	NA	NA	NA	NA	Buskey et al. [22]
1991	1	Lower Laguna Madre	NA	NA	NA	NA	Describes possible predatory mechanism for red tide control and a small persistent patch in a ship channel; Buskey et al. [22]
1996	21	Texas coast south of Galveston to Mexico border	9/28/1996	10/22/1996	9/12/1996	10/18/1996	
1997	11	Texas coast south of Galveston to Mexico border	9/25/1997	1/29/1998	9/18/1997	10/8/1997	
1998	2	NA	NA	NA	NA	NA	Description of a red tide conference and potential effects of red tide on National Seashore visitor attendance
2000	12	Texas coast south of Galveston to Mexico border	9/21/2000	10/24/2000	9/24/2000	10/24/2000	Confirmed by Cheng et al. [47]
2001	2	Nueces Estuary	12/20/2001	1/22/2002	NA	NA	
2002	1	NA	NA	NA	NA	NA	History of Corpus Christi that includes red tide of 1996
2005	8	Texas Coastal Bend and south	10/4/2005	12/19/2005	9/16/2005	9/16/2005	
2006	8	Texas Coastal Bend and south	10/2/2006	12/5/2006	10/3/2006	10/19/2006	
2009	15	Texas Coastal Bend and south	10/15/2009	12/31/2009	10/10/2009	12/19/2009	
2010	1	NA	NA	NA	NA	NA	Single article describing a *Dinophysis* bloom
2011	11	Texas Coast	10/7/2011	1/27/2012	10/7/2011	11/24/2011	
2012	4	Texas Coast	9/26/2012	9/26/2012	9/26/2012	9/26/2012	
2015	7	Texas Coast	10/2/2015	10/18/2015	9/6/2015	10/2/2015	Mentions co-occurring Trichodesmium bloom and how it may relate to red tide
2016	3	Texas Coastal Bend and south	9/10/2016	10/12/2016	9/7/2016	10/12/2016	Two articles earlier in the year mention previous red tides in another context

Qualitative assessment of these articles suggests that reporters were generally aware of red tides. For example, in the newspaper articles prior to 1996, 11 articles describe a red tide occurring elsewhere, regardless of the state of red tides in Texas coastal waters. Furthermore, a majority (75%) of the articles prior to 1996 referenced interviews with local, state, and/or academic scientists, strengthening the veracity of the reports. The 25% that did not cite someone from the scientific community tended to consist of articles mentioning red tide in another context (e.g. tourism dollars lost, efforts to help the economy, town festivals). There were also two occasions when red tide reports indicated that the causative agent was an organism other than *K*. *brevis* and one occasion where discolored water was found to be an oil drilling fluid spill.

When comparing the duration data derived from the newspaper to the duration as derived from the NOAA and TXHD records at three abundance thresholds, it was apparent that the publishing cycle and/or the occurrence of other newsworthy events were not likely to limit reporting of red tides. In the Nueces Estuary at the 5,000 cells L^-1^ and 10,000 cells L^-1^ thresholds, the duration tended to be shorter based on newspaper articles than based on cell counts (46.46 ± 40.94 vs 65.83 ± 40.75 and 62.75 ± 38.43; S2 Table). When duration was compared using the 100,000 cells L^-1^ threshold, the duration was more similar between the two records (46.46 ± 40.94 vs 46.17 ± 32.90; S2 Table). This suggests that duration may be underestimated for the Nueces Estuary in the newspaper data at low cell abundances (<100,000 cells L^-1^). In the Coastal Zone duration tended to be longer on average based on newspaper articles than based on cell counts at all thresholds compared (29.00 ± 21.29 vs 24.55 ± 31.79, 25.8 ± 31.82, 12.50 ± 24.53; S2 Table). In the Nueces Estuary, this may be indicative of remnant populations persisting in localized, poorly flushed regions of the estuary as has been hypothesized by others [23]. The duration estimates reported in newspapers may differ from those from agency-based data because of different response triggers. TXHD sampling efforts are initiated in response to red tide sightings and are concentrated in areas of shellfisheries whereas the NOAA dataset was a compilation of multiple sources [50], likely with different research objectives goals (i.e. toxin production, life cycle, ecophysiology). This is reflected by the fact that the NOAA dataset is more frequently the source of corroborating data in the Coastal Zone where TXHD is more frequently the corroborating source in the Nueces Estuary. Since agency-based sampling occurred in response to red tide sightings [50] the newspaper was likely to report a similar or earlier start date, as was observed in 7 out of 11 occurrences in Nueces Estuary and 6 out of 10 in the Coastal Zone. The 2009 red tide provides an example of the effects of differing temporal and spatial sampling efforts. The duration derived from the TXHD record is longer than that derived from the NOAA dataset in the Nueces Estuary, but the reverse is true in the adjacent Coastal Zone (S2 Table).

### 3.2 Trends in bloom presence/absence

Results showed a statistically significant increase in the frequency of red tides for the Nueces Estuary and the Coastal Zone (Table 2). Explanation of the Odds Ratio and Nagelkerke pseudo R^2^ value can be found in Methods section 2.2.1.

**Table 2:** Summary information for the logistic regression of red tide occurrence vs year (year-only model) for each region.

Geographic Area	Estimate	Estimate Standard Error	95% Confidence Intervals	p-value	Odds Ratio	Nagelkerke Pseudo-R^2^
Nueces Estuary	0.06	0.02	0.02, 0.10	0.003	1.06	0.24
Coastal Zone from Port O’Connor to Land Cut	0.07	0.02	0.02, 0.12	0.002	1.08	0.29

Additionally, Pettitt’s test revealed a significant change in red tide frequency occurring around 1995 in the Nueces Estuary and the Coastal Zone (Table 3).

**Table 3:** Results of change point analysis (Pettitt’s Test), where p ≤ 0.5 is significant.

Geographic Area	Time Point	K_T_ Statistic	p-value
Nueces Estuary	1995	387	0.049
Coastal Zone from Port O’Connor to Land Cut	1995	347	0.101

For the coastal zone segment, there were no explanatory models that performed better than the year-only model and the analysis concluded there. The explanatory model for the Nueces Estuary included salinity, ENSO, and NAO (Table 4). ENSO and NAO were negatively related to red tide occurrence while salinity was positively related, indicating that higher salinity, negative ENSO phase, and negative NAO phase are more likely to correspond with red tide presence. The models presented above met all quality controls.

**Table 4:** Results from final logistic regression model explaining red tide occurrence chosen for the Nueces Estuary.

Geographic Area	Explanatory Variables	Estimates	Estimate Standard Error	95% Confidence Interval	Odds Ratio	Nagelkerke Pseudo-R^2^
Nueces Estuary	ENSO	-1.52	0.91	-3.56, -0.11	0.22	0.50
	NAO	-3.54	1.58	-7.28, -0.83	0.03	
	salinity	0.54	0.24	0.17, 1.14	1.72	

### 3.3 Trends in bloom duration

**Fig 2:**
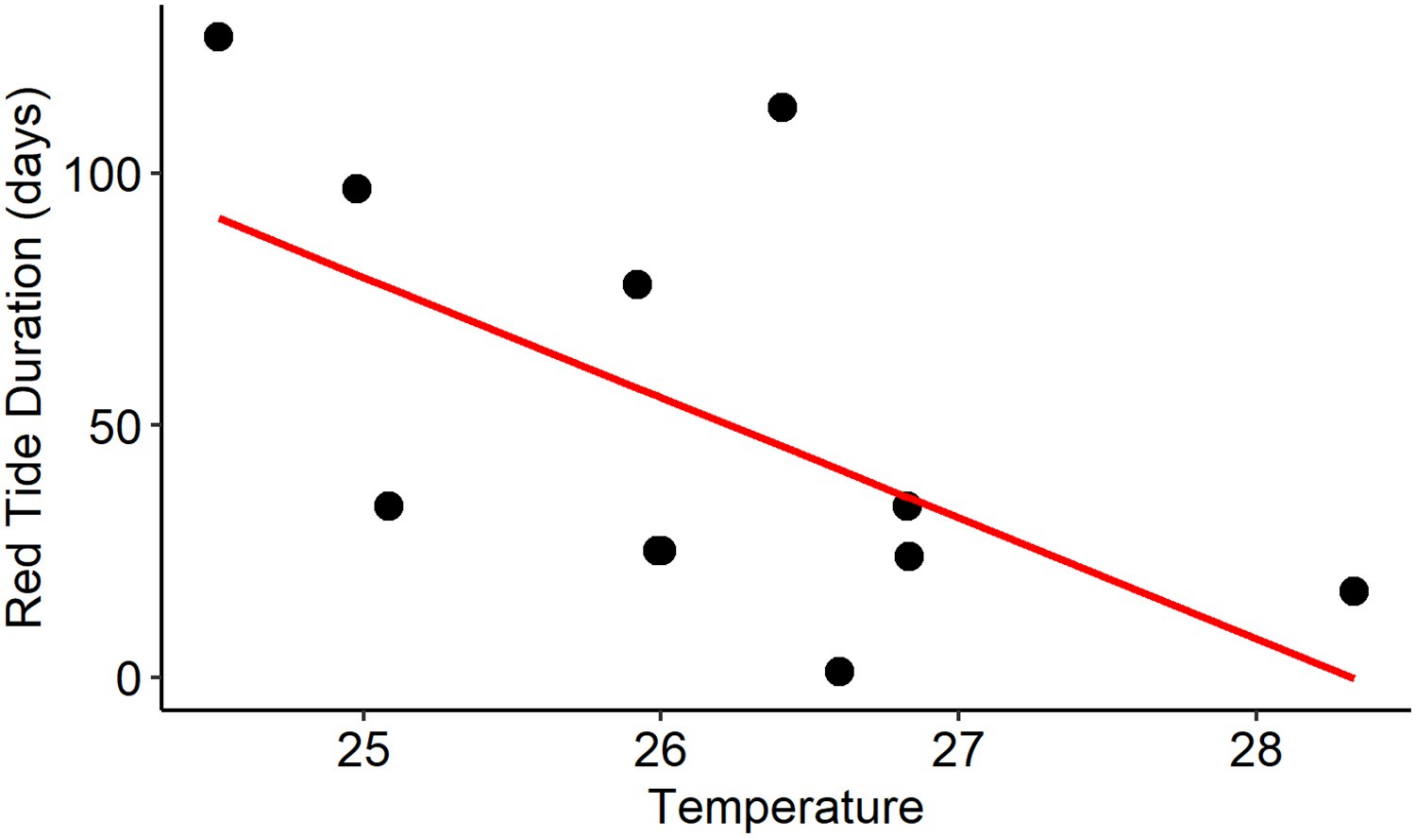
Red tide duration in the Nueces Estuary plotted against average fall temperatures (°C). Fall temperatures were calculated as the average of all temperatures recorded in the Texas Parks and Wildlife trawl dataset in the Nueces Estuary from August through November of each year (1982–2015). The red line represents the linear regression model fit.

The year-only linear regression models (year vs. red tide duration) for the Nueces Estuary and Coastal Zone were not significant, indicating that there was no change in red tide duration over time. The duration of red tides in the Nueces Estuary ranged from 1 to 127 days, with an average of 42 days and a median of 25 days. For the Coastal Zone segment, the duration of red tides ranged from 1 to 71 days, with an average and median of 24 days. For the Nueces Estuary, an explanatory model including temperature indicated a significant negative relationship between red tide duration and temperature (Fig 2). This model had an acceptable fit, was significantly different than the null model (α = 0.05, p = 0.03), and had an R^2^ of 0.34. For the Coastal Zone segment, there were no explanatory models that passed quality control.

### 3.4 Environmental conditions associated with bloom stages

For the full dataset with all stages, the final logistic regression model included average daily temperature and average daily wind speed with year and month as random factors. The model was significantly different than the null model (α = 0.01, p = < 0.001) and had a Nagelkerke’s pseudo R^2^ of 0.46 (Table 5). The explanatory model for the dataset that included B and D stages had daily average temperature with year and month as random factors. The model was significantly different than the null model (α = 0.01, p = < 0.001) and had a Nagelkerke’s pseudo R^2^ of 0.75. The explanatory model for the dataset with D and A stages included average daily wind speed with year and month as random factors. The model was significantly different than the null model (α = 0.01, p = < 0.001) and had a Nagelkerke’s pseudo R^2^ of 0.64.

**Table 5:** Summary information for the final three logistic regression models chosen to explain daily presence/absence of *K*. *brevis* red tides in Nueces Estuary.

Dataset	Explanatory Variables	Estimates	Estimate Standard Error	95% Confidence Interval	Odds Ratio	Nagelkerke Pseudo-R^2^
All Stages; B, D, A^a^	Temperature	-0.12	0.03	-0.19, -0.06	0.88	0.46
	Wind Speed	-0.12	0.07	-0.25, 0.01	0.89	
Stages B and D	Temperature	-0.52	0.09	-0.74, -0.34	0.59	0.75
Stages D and A	Wind Speed	-0.30	0.11	-0.53, -0.09	0.74	0.64

^a^ Stage B indicates the period of 14 days before a red tide, D indicates the period of red tide presence, and A indicates the period of 14 days after a red tide.

## 4. Discussion

HABs are influenced by human activity as well as natural climate oscillations and longer-term anthropogenic climate change [5, 13, 51]. One of the challenges in understanding long-term trends in a HAB of interest is the availability of data documenting its occurrence and duration, and that of key environmental drivers [13, 31]. The field of marine historical ecology has provided strong evidence for the use of non-traditional data sources in the assessment of historical conditions and trends [32, 33]. Here, datasets of red tide presence/absence and duration were generated using archived newspaper articles from the *Corpus Christi Caller Times*. Quantitative comparisons with modern (1996–2016) agency-based cell counts, as well as a qualitative assessment of the early record (1955–1996), provided ample support for the use of this source. A bias evident in many long-term datasets, especially those from non-traditional sources, is increased reporting due to increased awareness rather than true increases in the occurrence of events, HAB or otherwise. Articles from the early record (1955–1996) that referenced red tides from other geographical areas, listed warning signs and potential impacts of red tides, provided follow-up reports when discolored water was something other than *K*. *brevis*, and continued coverage on damages sustained during a previous red tide, are an indication of general awareness of red tides and interest in them by the news media. The advantages of using this non-traditional data source were two-fold: 1) it allowed for a quantitative assessment of trends in red tide occurrence from a data poor region (Texas coast, western Gulf of Mexico), and 2) it allowed us to identify climatological drivers (i.e. ENSO) as well as local environmental factors that are important for bloom initiation and demise in an estuarine setting, representing one of the first studies to do so.

The Nueces Estuary and adjacent Coastal Zone appear to have experienced a significant increase in the frequency of red tides between 1955 and 2016. In both locations, change point analysis indicated that the change from less to more frequent red tides occurred in approximately 1995. These findings agree with previous qualitative discussion of increases in red tide frequency in western Gulf of Mexico coastal waters [1, 23]. The assessment of the newspaper record leads us to conclude that these observed increases are not attributable to increased reporting in the recent record due to increased awareness. Five of the eight red tides reported in the newspaper prior to 1996 were corroborated by scientific publications and 75% of the articles written during this time referenced interviews with scientists suggesting consistent awareness of red tides among two time periods. This study examined the factors driving observed increases in red tide frequency and used that information to develop testable hypotheses with a goal of refining monitoring and forecasting approaches for *Karenia brevis* red tides.

In the Coastal Zone adjacent to the Nueces Estuary, explanatory modeling indicated that no combination of the environmental variables could explain red tide occurrence better than year alone. The finding that neither large-scale climate variability nor local conditions were important in explaining red tide occurrence aligns well with what is known about transport induced bloom initiation. For example, Thyng et al. [18] describe the importance of downcoast winds in determining whether cells are transported from offshore to the Texas coast, and suggest that interannual variability in wind speed/direction are important considerations in whether a bloom will develop or not in a given year.

Within the Nueces Estuary, salinity (positive), ENSO (negative), and NAO (negative) were important for explaining red tide occurrence, with a pseudo R^2^ of 0.50. The positive relationship between red tide occurrences and salinity is consistent with prior laboratory work on the physiological tolerances of *K*. *brevis*, which showed maximum growth rates at salinities of ~30–35, and decreasing growth rates at lower salinities [38, 52]. Additionally, analysis of long-term field data in Florida coastal waters indicated that only 3% of samples that were “positive” for *K*. *brevis* had salinity ≤ 24 [14]. Bugica et al. [34] recently demonstrated significant increases in salinity in the Nueces Estuary and other Texas Coastal Bend estuaries over the past 20–30 years due to damming and increased human demands on water resources. This trend is likely to continue with projected population growth in Texas coastal counties (Texas State Data Center, http://txsdc.utsa.edu/Data/TPEPP/Projections/Index.aspx) and warmer and drier conditions expected in the western Gulf of Mexico under changing climate pressures [35, 36]. Understanding how anthropogenic activities are affecting salinity regimes in these estuarine systems will be critical for assessing potential future frequency of red tides.

Positive ENSO phase is associated with increased rainfall on the Texas coast, as are lower salinities in estuaries [53]. This relationship between ENSO, rainfall, and salinity on the Texas coast explains the negative relationship between ENSO and red tide occurrence in the Nueces Estuary, with El Niño (positive ENSO) events leading to lower salinities that are not ideal for *K*. *brevis*. Aside from ENSO, the NAO was also related to red tides, exhibiting a negative relationship with red tide occurrence in the Nueces Estuary. A study by Parazoo et al. [54] examined precipitation extremes and documented that periods of strongly negative NAO amplified drought conditions in Texas, which would lead to higher salinities in estuaries and conceivably be favorable to *K*. *brevis*. This is consistent with our findings of a negative relationship between NAO and red tide occurrence in the Nueces Estuary. In other words, the negative phase of the NAO (drought, high salinity) would equate to greater likelihood of red tide occurrence.

A final consideration for the relationship between climate variability and red tide occurrence is the global regime shift that occurred in the mid-1990s and involved the NAO, the Atlantic Meridional Overturning Circulation, the Subpolar Gyre, the Atlantic Multidecadal Oscillation, and the Pacific Decadal Oscillation [55, 56]. The change point analysis conducted here coincides with this global regime shift, suggesting that either the relationship between NAO and red tide is merely coincidental, or that there are as yet unknown teleconnections between Atlantic modes of climate variability and the western Gulf of Mexico. Further work to understand how and at what time scale(s) these modes of climate variability individually and collectively influence circulation, temperature, and precipitation in this region is warranted.

Tester et al. [23] suggest that while circulation and transport are critical for bloom development along the Texas coast, conditions within estuaries (poorly flushed, high salinity) may maintain seed or remnant populations of *K*. *brevis* prolonging bloom conditions in estuaries. Therefore, understanding factors influencing bloom dynamics in the estuary will be critical in assessing risk to coastal waters of the western Gulf of Mexico now and in the future. No significant change in the duration of red tides was detected in the Nueces Estuary though temperature was negatively correlated with the duration of red tides (α = 0.05; p = 0.058). The relationship between temperature and red tide duration may be related to the physiological requirements of *K*. *brevis*, which has an optimum temperature range between 22°C and 28°C [9]. Magaña and Villareal [38] demonstrated highest *K*. *brevis* growth rates in cultures at salinities of 30 and 35 and temperatures of 20 and 25°C. They also found that their *K*. *brevis* cultures (native to S. Texas) could not be acclimated to temperatures greater than 30°C. Errera et al. [57] also demonstrated significantly lower growth rates of *K*. *brevis* cultures at 30°C relative to 25°C. Despite the borderline significance seen here, our results offer further support for the role of temperature in the daily red tide presence/absence analyses.

To investigate climatic conditions that are associated with the time periods preceding and following a bloom relative to those during a bloom, daily red tide presence/absence was modeled using daily weather conditions. The results presented and conclusions drawn here should be considered a first step towards furthering understanding of factors that facilitate bloom demise in estuarine waters. Starting with the model for all three stages (before, during, and after), air temperature and wind speed were negatively related to red tide presence. In the before bloom/during bloom model, air temperature was also negatively related to red tide presence and the effect was much larger than in the “all stages” model. This indicates that high (i.e., summer-like) temperatures are detrimental to red tides, specifically to the timing of their initiation. This finding is also consistent with knowledge on the seasonality of red tides in Texas. Not only is regional circulation conducive to transport of *K*. *brevis* biomass from offshore to in- and nearshore during the fall in Texas [17–19] but fall water temperature is typically well within the physiological optimum range of *K*. *brevis* (24°C– 28°C). For example, the average summer water temperature in the Nueces Estuary during the period of 1982–2015 (this study) was 29.3 ± 1.2 while the average fall water temperature was 25.8 ± 1.2. This lends further support to the hypothesis that cooler temperatures in fall are important in supporting red tide initiation and maintenance.

The only environmental variable of importance in the during bloom/after bloom model was wind speed, which was negatively related to red tide presence and accounted for greater than half (pseudo R^2^ = 0.64) of the variation between red tide presence and absence. Abrupt decreases in temperature and high turbulence associated with the passage of cold fronts have been suggested to be important in bloom decline based on anecdotal accounts of bloom dissipation, experimental evidence of decreased growth at sub-optimal temperatures, and field observations of lysed cells and aerosolized brevetoxin due to crashing waves [6, 9, 38]. Our finding agrees with the hypothesis that frontal passages and associated increased wind speeds and turbulence are likely critical in ending a red tide. However, they do not support a role for water temperature in bloom decline. The correlation with decreasing temperatures at the start of a bloom but lack of correlation with temperature at the end of blooms suggests that *K*. *brevis* may be better equipped to handle physiological stress from temperatures lower than optimum rather than higher. Culture and field studies have shown tolerance of *K*. *brevis* to temperatures much lower than optimum (~7°C vs. ~20°C/22°C) whereas the difference between highest temperature tolerated and the upper limit optimum (~32°C vs. ~ 23°C/28°C) is much smaller [20]. This supports our conclusion that *K*. *brevis* may handle low temperature stress more effectively than high temperature stress. When considered along with findings from laboratory-based studies on *K*. *brevis* temperature optima, results presented here suggest that future increases in summer-fall temperatures associated with anthropogenic climate change have the potential to delay the initiation of red tides, while increases in winter temperatures may act to delay the demise. Assuming that other environmental conditions (i.e. light, wind, salinity) are adequate for survival and growth of red tides, this could lead to scenarios where the window for red tides is shortened (if starting later in year), stays the same (if starting later but ending later), or lengthened (if starting later but extending much longer than normal).

## 5. Conclusion

Results show that red tides have been increasing in frequency on the Texas coast over the past 60 years, necessitating a better understanding of the environmental factors driving red tide occurrence. A recent assessment of water quality trends on the Texas coast only found clear signatures of eutrophication (high and/or increasing chlorophyll, nutrients) in two estuarine complexes (Baffin Bay-Upper Laguna Madre, Galveston Bay), although some evidence of eutrophication was found in smaller sub-estuaries and isolated regions of the larger estuaries [34]. In the Nueces Estuary, Bugica et al. [34] found increasing orthophosphate concentrations at five of nine sites in the system, but both ammonium and nitrate showed a long-term decrease throughout the estuary, and three of nine study sites showed decreasing chlorophyll levels. The lack of evidence for eutrophication argues against the hypothesis that it is a leading cause of increases in the frequency of red tides in the Nueces Estuary.

In contrast to the general lack of evidence for widespread eutrophication in the Nueces Estuary, Bugica et al. [34] found that salinity increased over time at all nine study sites in the system. The strong relationship between salinity and increasing frequency of red tides in the Nueces Estuary highlight the need to better understand the role of large-scale hydrologic forcing (rainfall, river discharge) on habitat suitability for *K*. *brevis* in Texas estuaries and nearshore coastal waters. Although not reported here, we also found evidence of increases in red tide frequency in other central Texas coast estuaries where long-term increases in salinity were also observed (Tominack et al. unpubl. data) [34]. Long-term increases in salinity are linked to damming and growing human water demands in coastal watersheds over the past ~50 years [36]. Population and climate projections suggest that over the coming century, Texas will see additional increases in population and water demands, as well as a warmer and drier climate [35, 36]. This will likely lead to further increases in salinity in Texas estuaries, leading to conditions that are more similar to seawater and thus more hospitable to *K*. *brevis* [58]. Though freshwater inflow management in Texas has changed from resource- to ecosystem-based following the introduction of Senate Bill 3 in 2007 (https://www.twdb.texas.gov/surfacewater/flows/freshwater/index.asp), implications of freshwater inflow management have not considered red tide habitat suitability to date.

An additional implication of this study’s findings pertains to efforts aimed at forecasting red tide blooms. Early warning detection (days to weeks lead time) of red tide in the western Gulf of Mexico is already being done through automated cell imaging and counting [59] as well as satellite remote sensing [60, 61]. The strong relationships between ENSO/NAO, salinity and red tide occurrence seen here offer an opportunity for even longer lead times considering that ENSO forecasts are often produced many months in advance [62]. Lastly, though it is recognized that many other factors (i.e. nutrient availability, grazing, viral lysis) may play a role in bloom demise [6, 9, 29, 63], our investigation offers valuable insight into factors limiting the duration of blooms. Future research in estuarine systems should consider the use of targeted monitoring programs, Lagrangian drifters, and/or modelling efforts to quantify the relative importance of environmental conditions (i.e. temperature, wind speed and direction) in determining conditions that may prolong active/remnant red tides or lead to their demise.

## Supporting information

S1 Fig Comparison of air and water temperature (°C) at Packery Channel in the Nueces Estuary. Data were obtained from https://tidesandcurrents.noaa.gov, station number 8775792, for the time period of August 2012 thru October 2018.
(TIF)

S1 Table Fall seasonal average water temperature (°C) ± standard deviation, salinity ± standard deviation, and number of observations for Texas Parks and Wildlife trawl dataset. Seasonal averages are comprised of values from August thru November of each year.
(DOCX)

S2 Table Comparison of red tide duration (days) from newspaper reports with cell counts data from the Texas Health Department (TXHD) and NOAA Harmful Algal Bloom Observation Study (HABSOS) [30]. A 2000 red tide in the Coastal Zone during the same period as in the newspaper accounts was confirmed by Magaña et al. [1] and Cheng et al. [40].
(DOCX)
