# Supplementary figures and images for: An assessment of trends in the frequency and duration of Karenia brevis red tide blooms on the South Texas coast (western Gulf of Mexico)
Source: PLoS One. 2020 Sep 18;15(9):e0239309. doi: 10.1371/journal.pone.0239309 (PMC7500669; doi:10.1371/journal.pone.0239309)

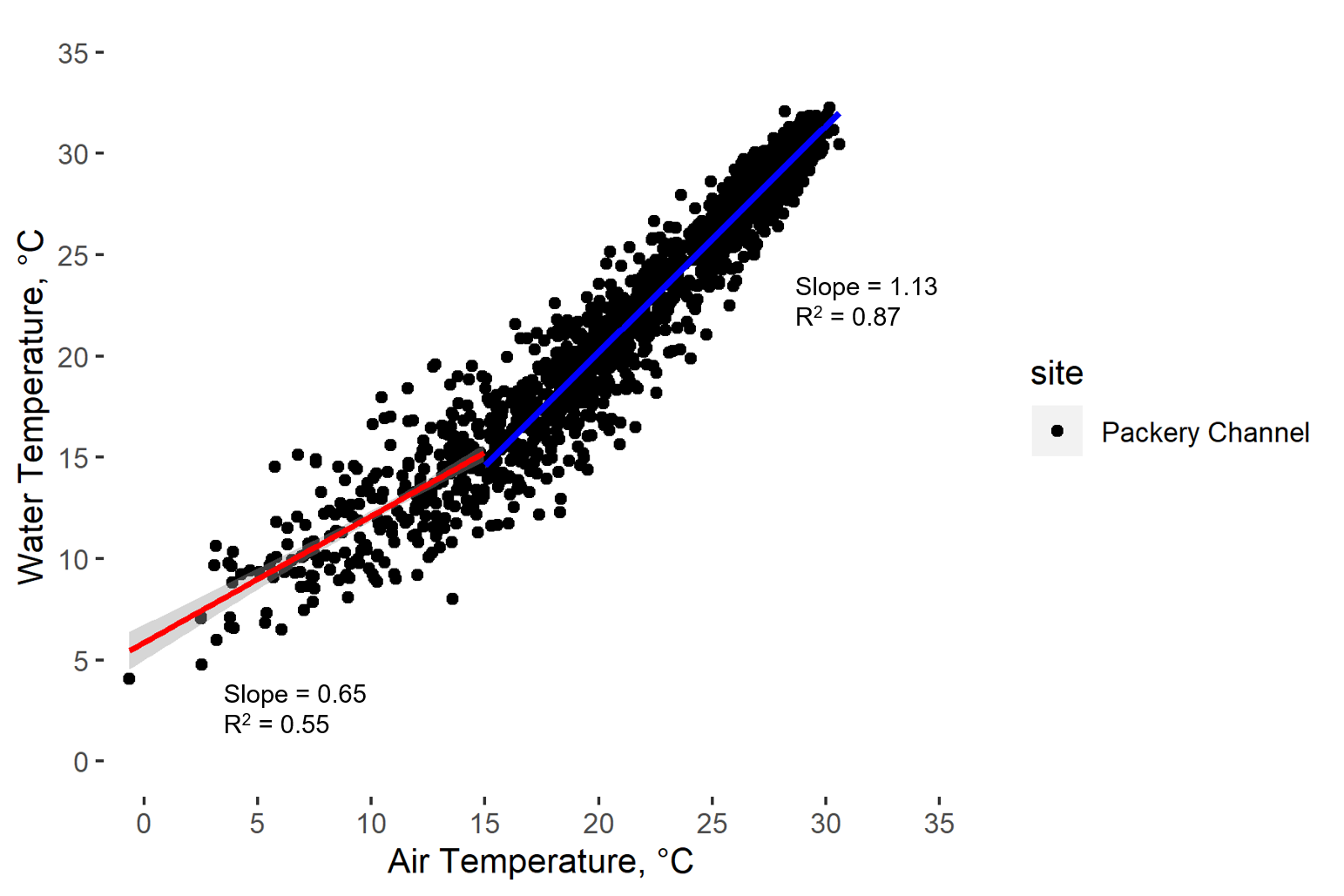

Supplement: S1 Fig — Data were obtained from https://tidesandcurrents.noaa.gov, station number 8775792, for the time period of August 2012 thru October 2018. (TIF) [file pone.0239309.s001.tif]
