# Supplementary material for: An assessment of trends in the frequency and duration of Karenia brevis red tide blooms on the South Texas coast (western Gulf of Mexico)
Source: PLoS One. 2020 Sep 18;15(9):e0239309. doi: 10.1371/journal.pone.0239309 (PMC7500669; doi:10.1371/journal.pone.0239309)
Supplement: S1 Table — Seasonal averages are comprised of values from August thru November of each year. (DOCX) [file pone.0239309.s002.docx]

| Year | Nueces Estuary | | | Coastal Zone Port O'Connor to Land Cut | | |
| --- | --- | --- | --- | --- | --- | --- |
|  | Temperature (°C) | Salinity | No. Observations | Temperature (°C) | Salinity | No. Observations |
| 1982 | 25.61 ± 4.86 | 33.46 ± 2.27 | 116 |  |  |  |
| 1983 | 26.2 ± 3.31 | 28.73 ± 2.39 | 114 |  |  |  |
| 1984 | 24.53 ± 4.44 | 34.69 ± 4 | 114 |  |  |  |
| 1985 | 26.59 ± 3.61 | 32.95 ± 4.23 | 80 | 26.64 ± 2.62 | 31.96 ± 2.55 | 64 |
| 1986 | 24.98 ± 6.2 | 35.95 ± 6.36 | 80 | 25.67 ± 2.97 | 32.82 ± 3.71 | 64 |
| 1987 | 24.72 ± 4.47 | 32.59 ± 4.2 | 80 | 25.18 ± 2.95 | 36.71 ± 2.11 | 64 |
| 1988 | 26.6 ± 3.62 | 36.67 ± 2.16 | 80 | 26.45 ± 3 | 33.93 ± 2.72 | 64 |
| 1989 | 25.82 ± 3.31 | 36.44 ± 5.03 | 80 | 25.88 ± 3.38 | 30.62 ± 3.91 | 64 |
| 1990 | 26.04 ± 4.01 | 33.93 ± 5.87 | 80 | 26.9 ± 2.8 | 33.73 ± 1.67 | 64 |
| 1991 | 25.06 ± 5.83 | 33.6 ± 3.92 | 80 | 25.26 ± 4 | 34.15 ± 3.32 | 64 |
| 1992 | 23.79 ± 3.97 | 30.24 ± 2.95 | 104 | 25.7 ± 3.1 | 33.98 ± 4.45 | 64 |
| 1993 | 25.16 ± 4.85 | 33.49 ± 3.41 | 104 | 24.95 ± 3.93 | 35.32 ± 3.91 | 64 |
| 1994 | 26.42 ± 2.65 | 33.91 ± 3.46 | 104 | 26.28 ± 2.76 | 32.01 ± 3.42 | 64 |
| 1995 | 26.31 ± 4.64 | 33.24 ± 3.12 | 104 | 26.12 ± 4.17 | 31.58 ± 1.61 | 56 |
| 1996 | 25.98 ± 3.35 | 38.06 ± 3.12 | 80 | 25.75 ± 3.02 | 34.39 ± 3.13 | 64 |
| 1997 | 24.51 ± 5.57 | 30.19 ± 5.4 | 80 | 24.73 ± 4.52 | 32.82 ± 2.92 | 64 |
| 1998 | 26.37 ± 3.58 | 31.46 ± 8.59 | 80 | 26.11 ± 3.12 | 32.62 ± 4.03 | 64 |
| 1999 | 26.33 ± 3.57 | 31.71 ± 2.91 | 80 | 26.64 ± 2.76 | 33.93 ± 2 | 64 |
| 2000 | 25.08 ± 5.43 | 37.31 ± 3.04 | 80 | 24.17 ± 3.25 | 33.87 ± 1.85 | 64 |
| 2001 | 26.82 ± 3.3 | 33.9 ± 5.24 | 80 | 26.45 ± 2.69 | 33.39 ± 3.28 | 64 |
| 2002 | 26.16 ± 4.12 | 19.02 ± 7.72 | 80 | 26.35 ± 3.15 | 30.65 ± 3.97 | 64 |
| 2003 | 26.89 ± 3.53 | 27 ± 7.69 | 80 | 26.65 ± 1.83 | 32.6 ± 3.66 | 64 |
| 2004 | 26.72 ± 3.77 | 27.68 ± 3.66 | 80 | 27.85 ± 2.76 | 32.97 ± 2.5 | 64 |
| 2005 | 26.83 ± 4.51 | 34.21 ± 2.36 | 80 | 27.43 ± 3.53 | 32.18 ± 2.2 | 64 |
| 2006 | 26 ± 3.5 | 35.45 ± 2.91 | 80 | 26.98 ± 2.47 | 34.48 ± 1.5 | 64 |
| 2007 | 25.62 ± 5.51 | 20.38 ± 4.93 | 80 | 27.62 ± 2.82 | 30.27 ± 2.57 | 64 |
| 2008 | 25.85 ± 3.45 | 33.4 ± 1.89 | 80 | 25.16 ± 2.34 | 32.18 ± 2.88 | 64 |
| 2009 | 25.92 ± 4.08 | 35.45 ± 2.83 | 80 | 26.19 ± 2.74 | 31.58 ± 2.63 | 64 |
| 2010 | 26.14 ± 4.68 | 27.9 ± 4.63 | 80 | 27.23 ± 2.52 | 31.99 ± 2.37 | 64 |
| 2011 | 26.4 ± 3.32 | 39.51 ± 2.1 | 80 | 26.93 ± 2.57 | 35.16 ± 1.94 | 64 |
| 2012 | 26.6 ± 4.37 | 39.58 ± 2.49 | 60 | 26.16 ± 3.22 | 34.08 ± 3.02 | 48 |
| 2013 | 21.26 ± 1.87 | 33.6 ± 1.96 | 20 | 21.61 ± 0.46 | 30.3 ± 1.92 | 16 |
| 2014 | 25.86 ± 5.49 | 38.84 ± 2.31 | 80 | 26.14 ± 3.71 | 35.05 ± 1.86 | 64 |
| 2015 | 28.32 ± 1.75 | 31.77 ± 3.57 | 60 | 27.87 ± 1.85 | 31.83 ± 3.32 | 48 |
