# Supplementary material for: An assessment of trends in the frequency and duration of Karenia brevis red tide blooms on the South Texas coast (western Gulf of Mexico)
Source: PLoS One. 2020 Sep 18;15(9):e0239309. doi: 10.1371/journal.pone.0239309 (PMC7500669; doi:10.1371/journal.pone.0239309)
Supplement: S2 Table — A 2000 red tide in the Coastal Zone during the same period as in the newspaper accounts was confirmed by Magaña et al. [1] and Cheng et al. [40]. (DOCX) [file pone.0239309.s003.docx]

| Nueces Estuary | | | | | |
| --- | --- | --- | --- | --- | --- |
| Year | Newspaper | 5,000 cells L^-1^ | 10,000 cells L^-1^ | 100,000 cells L^-1^ | Scientific Record |
| 1996 | 25 | 43 | 43 | 15 | TXHD |
| 1997 | 127 | 40 | 40 | 40 | TXHD |
| 1998 |  |  |  |  |  |
| 1999 |  |  |  |  |  |
| 2000 | 34 | 25 | 25 | 25 | TXHD |
| 2001 | 34 | 109 | 101 | 101 | TXHD |
| 2002 |  |  |  |  |  |
| 2003 |  |  |  |  |  |
| 2004 |  |  |  |  |  |
| 2005 | 24 | 77 | 60 | 60 | TXHD |
| 2006 | 25 | 65; 37 | 65; 37 | 17; 17 | TXHD; HABSOS |
| 2007 |  |  |  |  |  |
| 2008 |  |  |  |  |  |
| 2009 | 78 | 153; 84 | 147; 78 | 85; 64 | TXHD; HABSOS |
| 2010 |  |  |  |  |  |
| 2011 | 113 | 87 | 87 | 87 | HABSOS |
| 2012 | 1 |  |  |  |  |
| 2013 |  |  |  |  |  |
| 2014 |  |  |  |  |  |
| 2015 | 17 | 69 | 69 | 42 | TXHD |
| 2016 | 33 | 1 | 1 | 1 | TXHD |
| Mean ± Standard Deviation | 46.46 ± 40.94 | 65.83 ± 40.75 | 62.75 ± 38.43 | 46.17 ± 32.90 |  |

| Coastal Zone from Port O’Connor to Land Cut | | | | | |
| --- | --- | --- | --- | --- | --- |
| Year | Newspaper | 5,000 cells L-1 | 10,000 cells L^-1^ | 100,000 cells L^-1^ | Scientific Record |
| 1996 | 37 | 2 | 2 | 2 | TXHD |
| 1997 | 20 | 20 | 20 |  | TXHD |
| 1998 |  |  |  |  |  |
| 1999 |  |  |  |  |  |
| 2000 | 31 |  |  |  | Magaña et al. [1]; Cheng et al. [40] |
| 2001 |  |  |  |  |  |
| 2002 |  |  |  |  |  |
| 2003 |  |  |  |  |  |
| 2004 |  |  |  |  |  |
| 2005 | 1 | 8 | 2 |  | HABSOS |
| 2006 | 17 | 20 | 20 | 10 | HABSOS |
| 2007 |  |  |  |  |  |
| 2008 |  |  |  |  |  |
| 2009 | 71 | 1; 78 | 78 |  | TXHD; HABSOS |
| 2010 |  |  |  |  |  |
| 2011 | 49 | 93 | 88 | 62 | HABSOS |
| 2012 | 1 |  |  |  |  |
| 2013 |  | 20 | 20 | 1 | HABSOS |
| 2014 |  |  |  |  |  |
| 2015 | 27 | 27 | 27 | 1 | TXHD |
| 2016 | 36 | 1 | 1 | 1 | TXHD |
| Mean ± Standard Deviation | 29.00 ± 21.29 | 24.55 ± 31.79 | 25.80 ± 31.82 | 12.50 ± 24.53 |  |
